# The prevalence and risk factors of *Trichomonas vaginalis* in Wuhan and the Tibetan area, China: a two-center study

**DOI:** 10.1007/s00436-022-07726-x

**Published:** 2022-11-25

**Authors:** Xiaowu Zhu, Linlin Liu, Lamu Yixi, Yanan Yang, Yan Zhang, Zhen Yang, Huali Chen, Jinfeng Dong, Shouhua Yang

**Affiliations:** 1 Department of Gynecology and Obstetrics, Union Hospital, Wuhan, Hubei China; 2 Department of Gynecology and Obstetrics, Maternal and Child Health Hospital, Shannan, Tibet, China; 3 Department of Gynecology and Obstetrics, Maternal and Child Health Hospital, Yingcheng, Hubei China; 4 Department of Gynecology and Obstetrics, The Third People’s Hospital, Jianli, Hubei China

**Keywords:** *Trichomonas vaginitis*, Tibetan area, Bacterial vaginosis, Vulvovaginal candidiasis, Prevalence

## Abstract

*Trichomonas vaginalis* (*T. vaginalis*) infection is one of the most common sexually transmitted infections worldwide and is associated with several complications. However, the paucity of research regarding the prevalence of *T. vaginalis* infection in the Tibetan area limits control efforts. We aimed to evaluate the prevalence of *T. vaginalis* infection in the Tibetan area by a comparison with the prevalence of *T. vaginalis* in Wuhan city and to unveil the potential risk factors in the Tibetan area. This descriptive, cross-sectional study was conducted among adult women attending gynecology outpatient clinics in two public hospitals (one in Shannan city of Tibet and one in Wuhan city) in China in 2020. Data were retrieved from the medical record system and laboratory information management system, including *T. vaginalis* infection, bacterial vaginosis, and vulvovaginal candidiasis by wet mount microscopy or nucleic acid hybridization of vaginal secretions from patients. The associations of variables associated with *T. vaginalis* prevalence were quantified by odds ratios with 95% confidence intervals. The overall prevalence rates of *T. vaginalis* infection in the Tibetan area and Wuhan city were 20.94% and 2.84%, respectively. The statistically significant factors for the higher prevalence of *T. vaginalis* infection in the Tibetan area included tertiary educational status (AOR: 0.36 [95% CI: 0.16–0.81]), yearly family income > ¥100,000 (AOR: 0.48 [95% CI: 0.26–0.91]), clinical symptoms (AOR: 4.58[95% CI: 2.32–9.04]), and III–IV grade vaginal cleanliness (AOR: 29.71 [95% CI: 3.95–223.56]) in the multivariate logistic analysis. Interventions targeting improved living standards as well as women’s educational level and promoting reproductive hygiene habits are recommended to contribute to the reduction in *T. vaginalis* infection in the Tibetan area.

**Supplementary Information:**

The online version contains supplementary material available at 10.1007/s00436-022-07726-x.

## Introduction

*Trichomonas vaginalis* (*T. vaginalis*) infection, caused by a flagellated protozoan, is responsible for the most common curable sexually transmitted infection (STI) worldwide (Margarita et al. 2019; Workowski and Bolan 2015). *T. vaginalis* is recognized as one of the causative agents of infectious vaginitis (Schwebke and Burgess 2004), affecting an estimated 244.90 million cases in 195 countries (Collaborators 2018). Although up to 50% of female infections are completely asymptomatic, untreated *T. vaginalis* infection is well-established risk factor for vaginitis, cervicitis, pelvic inflammatory diseases, adverse outcomes of pregnancy, increased susceptibility to human papillomavirus (HPV) infections and cervical cancers, and even acquisition and transmission of human immunodeficiency virus (HIV) (Belfort et al. 2021; Blair et al. 2011; Bouchemal et al. 2017).

The prevalence estimates of *T. vaginalis* infection vary among different demographic groups and geographical regions. According to World Health Organization (WHO n.d.) reports, the global *T. vaginalis* infection rate was 5.30% in women, with the highest value of 11.70% in the African region and the lowest value of 1.60% in the European region (Rowley et al. 2019). In China, although there are no nationwide data on *T. vaginalis* prevalence, previous cross-sectional studies documented prevalence rates of 3.20%, 0.38%, 1.64%, and 7.44% in the southeastern (Fujian Province) (Chen et al. 2006), southern (Guangxi Province) (Lu et al. 2022), central (Henan Province) (Zhang et al. 2018), and northeastern (Jilin Province) (Li et al. 2008) areas of China, respectively. However, in clinical practice, we noticed that the prevalence of *T. vaginalis* infection appears to be higher in the Tibetan area than that reported by many regional population-based surveys in China (Chen et al. 2006; Dai et al. 2010; Fang et al. 2007; Li et al. 2008; Lu et al. 2022; Zhang et al. 2018). The situation creates public interests and health concerns in the area.

However, until now, relevant information for the high prevalence of *T. vaginalis* infection in the Tibetan area has remained sparse. Here, we conducted a cross-sectional study to highlight the difference in *T. vaginalis* prevalence between the Tibetan area and Wuhan city and then further analyzed potential associations between *T. vaginalis* prevalence and socioeconomic, behavioral, clinical, and microecological factors among the female population in the Tibetan area.

## Method

### Subjects

**Fig. 1:**
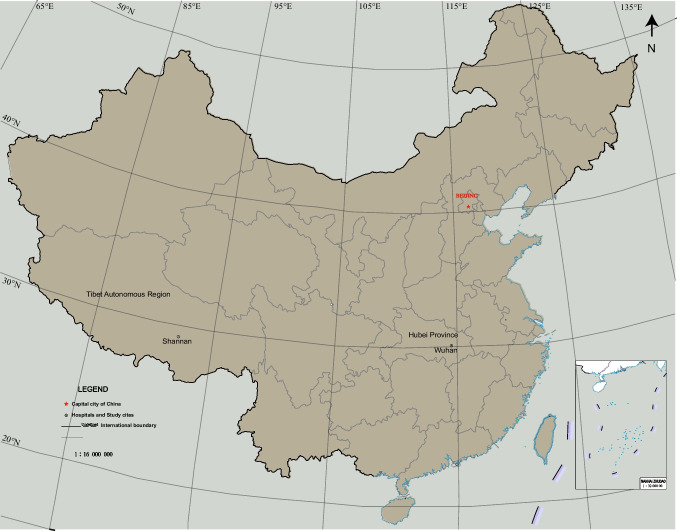
Map of China showing the two studied locations

An institutional-based, comparative cross-sectional survey was conducted to evaluate the prevalence of *T. vaginalis* infection among women treated in two hospitals, the Maternal and Child Health Hospital of Shannan city and Wuhan Union Hospital, with different geographical backgrounds. Shannan city (29°24′N, 91°77′E) of the Tibet Autonomous Region, located in northwest China, is approximately 3000 km from Wuhan city (30°60′N, 114°27′E) which is a provincial capital city in central China (Fig. 1). Survey data were obtained from clinical visits, outpatient medical record systems, and laboratory information management systems of the hospital in the Tibetan area (June 1 to October 15, 2020) and the hospital in Wuhan city (December 1 to December 30, 2020).

Women (aged 15 years and older) with reported sexual activity, who came to the gynecology and obstetrics outpatient clinic of the above hospitals, were included in the analyses during the data collection time, regardless of the reason for vaginal secretion examination that day. Subjects were excluded if they (1) had other serious complications, (2) had taken antibiotics, or (3) underwent vaginal treatments within 2 weeks prior to the visit. Data were provided to us from the institution after removing all subject identifiers.

Ethics approval for this study was obtained from the Ethics Committee of Wuhan Union Hospital and the Maternal and Child Health Hospital of Shannan city. Written informed consents were waived in view of the retrospective nature of the study. Informed oral consent was requested from the patients by institutional review board of the Maternal and Child Health Hospital of Shannan city.

### Data collection and diagnostic criteria

In Wuhan city, medical information (Spreadsheet 1) was extracted from medical chart records. Data in the Tibetan area (Spreadsheet 2) were originally abstracted through (voluntary) questionnaires as well as the outpatient medical record including socioeconomic factors (age, ethnic group, education, occupation, yearly family income, and marital status) and clinical and behavioral information (current pregnancy, gravidity, frequency of sex and contraception within the past 6 months, bath, frequency of vulva cleaning, smoking and alcohol habits, and symptoms). Women who had unusual vaginal discharge, itching, odor, irritations, or pelvic pain in the past week were included in the positive symptom group. The age and yearly family income groups were divided according to the median. Then, data related to the condition of the vulva, vagina, secretions, and cervix from gynecological examination records were collected and combined with other data.

Laboratory-based data relied on vaginal exudate samples collected by a sterile vaginal swab from the posterior fornix and the vaginal wall after examinations of the vagina and cervix with a speculum. In the Tibetan area, saline drops with the vaginal sample were smeared immediately on a clean glass slide with a cover slip and then examined microscopically at a magnification of × 40 for motile pear-shaped trophozoites within 10 min. This slide was also examined microscopically for vaginal cleanliness, clue cells, and hyphae or spores by qualified laboratory personnel. Then, the second vaginal swab was heat-fixed, gram-strained, and examined under a 100 × oil immersion objective for the diagnosis of bacterial vaginosis (BV) (Nugent et al. 1991). This slide was also observed under an oil immersion field microscope for the presence of mycelia or pseudohyphae. In Wuhan city, nucleic acid hybridization (Dessai et al. 2020) was performed by placing vaginal swabs into sterile sampling containers with Stuart’s medium and transporting them to the laboratory for detecting microbiological species.

For wet mount microscopy, positive vulvovaginal candidiasis (VVC) results were diagnosed microscopically by the presence of mycelia or pseudohyphae. A positive *T. vaginalis* result was determined by identification of pear-shaped and motile organisms with flagella and undulating membranes (Schwebke and Burgess 2004). A clinical positive diagnosis of BV was defined as a Nugent score of 7–10 (Nugent et al. 1991) or the presence of three out of four Amsel’s criteria (Amsel et al. 1983): homogenous vaginal discharge, pH > 4.5, clue cells > 20% on saline microscopy, and a positive “whiff test” result (a fishy odor on addition of 10% KOH to vaginal fluid). For nucleic acid hybridization, clinically significant amounts of DNA of *T. vaginalis* (5 × 10^3^ cells/ml), *Gardnerella* (2 × 10^5^ CFU/ml), and *Candida* species (1 × 10^4^ CFU/ml) were treated as positive results by the BD Affirm VPIII Microbial Identification Test Kit (Becton, Dickinson and Company, USA).

### Statistical analysis

All related data are summarized in Supplementary Spreadsheets and were analyzed using SPSS software (version 26). The independent *t* test or chi-square tests were used for continuous and categorical variables, respectively, to estimate statistical comparisons between two groups. To identify significant differences in subgroups of Tibetan data, the chi-square test or Fisher’s exact test was used in univariate analysis. A logistic regression model was performed in multivariate analysis including only *P* < 0.05 variables in univariate analysis. Figures were created and arranged using GraphPad Prism (version 8) and Adobe Illustrator 2022. *P* < 0.05 was considered statistically significant.

## Results

### Baseline patient characteristics

A total of 406 patients in the Tibetan area and 529 patients in Wuhan city were included in this study. Of the 529 patients in Wuhan, average age was 36.57 years (range 15 to 83 years), 96.03% were married or living with partner, and none were ethnic Tibetan. In the Tibetan area, the study sample consisted of 406 patients with mean ages of 34 years (range from 18 to 68 years), the majority of whom were Tibetan (89.90%), married (94.09%), and had a yearly family income ≤ 100,000 yuan (59.36%). Approximately one-third (31.28%) reported a primary or below education, and nearly four in ten (40.89%) were unemployed (farmers or herdsmen). Demographics of the patients are presented in Tables 1 and 2.

**Table 1:** Comparison of sociodemographic characteristics and prevalence of *T. vaginalis* in the Tibetan area and Wuhan city

Variable	Tibetan area	Wuhan city	*P* value^a^
	*N* = 406	*N* = 529	
Age (mean ± SD^b^, years)	34.24 ± 8.25	36.57 ± 11.80	< 0.01
Ethnic group, *n* (%)			< 0.01
Tibetan	365 (94.12)	0 (0.00)	
Others	41 (5.88)	529 (100.00)	
Married or living with partner, *n* (%)			0.17
Yes	382 (94.09)	508 (96.03)	
No	24 (5.91)	21 (3.97)	
At least one clinical symptom, *n* (%)			< 0.01
Yes	209 (51.48)	356 (67.30)	
No	197 (48.52)	173 (32.70)	
Trichomonas vaginalis, *n* (%)	85 (20.94)	15 (2.84)	< 0.01
Vulvovaginal candidiasis, *n* (%)	26 (6.40)	134 (25.33)	< 0.01
Bacterial vaginosis, *n* (%)	69 (17.00)	222 (41.97)	< 0.01

^a^*P* value shows the statistical significance as obtained from the independent *t* test and chi-square tests for continuous and categorical variables, respectively

**Table 2:** Univariate and multivariate analyses of factors associated with *T. vaginalis* infection in 406 women treated in the Tibetan area

Risk factor	n/N	TV positive (%)	Univariate		Multivariate^*^	
			χ^2^	*P* value	AOR (95% CI)	*P* value
Socioeconomic characteristics						
Age (years)			4.38	** < 0.05**		0.08
≤ 35	251/405	61 (24.30)			Ref	
> 35	154/405	24 (15.60)			0.61 (0.35–1.06)	
Ethnic group			2.11	0.15		
Tibetan	365/406	80 (21.92)				
Others	41/406	5 (12.20)				
Education level			18.79	** < 0.01**		** < 0.01**
Primary or below	127/406	32 (25.20)			Ref	
Secondary	148/406	42 (28.38)			1.17 (0.67–2.06)	0.58
Tertiary	131/406	11 (8.40)			0.36 (0.16–0.81)	0.01
Occupation			0.14	0.93		
Unemployed	166/406	35 (21.08)				
Employed/self employed	167/406	34 (20.36)				
Medical personnel	71/406	16 (22.54)				
Yearly family income (¥)			17.11	** < 0.01**		** < 0.05**
≤ 100,000	241/398	68 (28.22)			Ref	
> 100,000	157/398	17 (10.83)			0.48 (0.26–0.91)	
Marital status			0.00	0.99		
Single	24/406	5 (20.83)				
Married	382/406	80 (20.94)				
Clinical and behavioral factors						
Current pregnancy	62/405	13 (20.97)	0.00	0.96		
Gravidity			0.37	0.55		
≤ 1	101/406	19 (118.80)				
≥ 2	305/406	66 (21.64)				
Sexual behavior/week^a^			7.05	** < 0.05**		0.07
None	26/377	1 (3.85)			Ref	
≥ 1	161/377	32 (19.88)			6.28 (0.75–52.92)	0.91
< 1	190/377	49 (25.79)			9.20 (1.11–76.59)	0.04
Contraception			4.59	** < 0.05**		0.72
Condom	97/380	13 13.40)			0.87 (0.41–1.85)	
Others	283/380	67 (23.67)			Ref	
Bath^b^			0.39	0.82		
Sitz bath	105/406	19 (18.10)				
Shower bath	270/406	51 (18.89)				
Public bath	128/406	27 (21.09)				
Vulva cleaning/week			6.03	** < 0.05**		0.05
≥ 1	325/406	60 (18.46)			Ref	
< 1	81/406	25 (30.86)			2.00 (1.00–3.99)	
Tobacco use	14/401	5 (35.71)	1.91^**^	0.18		
Alcohol use	89/401	21 (23.60)	0.48	0.49		
Clinical symptom ^c^			44.22	** < 0.01**		** < 0.01**
Yes	209/406	71 (33.97)			Ref	
No	197/406	14 (7.11)			4.58 (2.32–9.04)	
Vaginal cleanliness^d^			31.40	** < 0.01**		** < 0.01**
I–II grade	99/406	1(1.01)			Ref	
III–IV grade	307/406	84 (27.36)			29.71 (3.95–223.56)	
VVC^e^	6/406	0(0.00)	7.36	** < 0.01**	0.00	1.00
BV^f^	69/406	17 (24.64)	0.69	0.41		

*Variables in the multivariate model were the only variables included in the univariate analysis with p < 0.05

**Fisher’s exact test

^a^Sexual behavior < 1/week was defined as sexual behavior more than once per year but less than once per week

^b^The sum more than the total due to multiple choices

^c^Clinical symptoms included itching, abnormal odor, vaginal discharge, abdominal pain, and frequent urination

^d^Vaginal cleanliness was graded based on coccus, epithelial cells, grading bacillus, and leukocytes per high magnification field (Yu et al. 2018)

^e^The diagnoses of VVC were based on mycelia or pseudohyphae observed in vaginal smears

^f^BV can be diagnosed by using the Amsel’s criteria or Nugent score

*N*, number; *AOR*, adjusted odds ratio; *95% CI*, 95% confidence interval; *Ref*, reference; *TV*, *Trichomonas vaginalis*; *VVC*, Vulvovaginal candidiasis; *BV*, bacterial vaginosis

### Prevalence of vaginitis in two regions

There was a higher prevalence of *T. vaginalis* infection but a lower prevalence of BV or VVC in the Tibetan area (all *P* < 0.01). As depicted in Table 1, statistically significant differences (*P* < 0.01) were detected in age and ethnic groups between the two regions, except for marital status (*P* > 0.05).

**Fig. 2:**
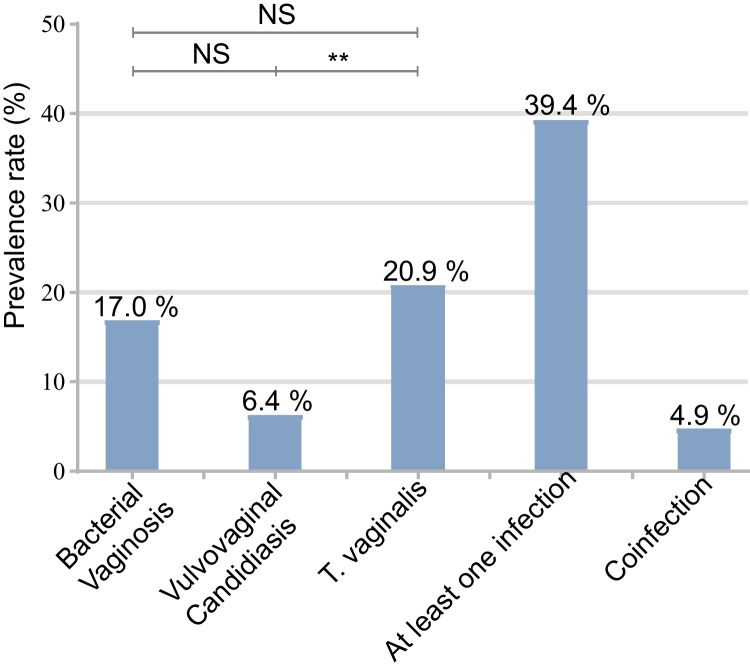
The prevalence of vaginitis infections in the Tibetan area (*N* = 406). There was a significant difference between *T. vaginalis* and VVC infection (*P* < 0.01). **P < 0.01; NS, no significance

In Wuhan city, the most common vaginal infection was BV (41.97%), 25.33% women had VVC, 2.84% *T. vaginalis* (Table 1)*. T. vaginalis* infection was the most common vaginal infection of the three in the Tibetan area (20.94%), following by BV (17.00%) and VVC (6.40%) (Fig. 2). On further analyses, we found a significant difference between the prevalence of *T. vaginalis* and VVC (*P* < 0.05), but there were no significant differences between BV and *T. vaginalis* or VVC (*P* > 0.05). At least one infection (BV, VVC or *T. vaginalis* infection) was found in 39.41% (160/406) of the women. The rate of coinfection was 4.93% (20/406): 17 subjects (4.19%) were coinfected with *T. vaginalis* and BV, and 3 subjects (0.74%) were coinfected with BV and VVC.

### Factors that influence T. vaginalis infection in Tibetan patients

Table 2 shows that the *T. vaginalis* prevalence varied among the subgroups. Women equal to or less than 35 years old had a somewhat higher prevalence (24.30%) of *T. vaginalis* infection (*P* < 0.05). Prevalence rates of 8.4% and 10.8% were observed in women with tertiary (college or higher) education and family income > 100,000 yuan/year, respectively; the rates increased when stratified women had less education or lower family income (*P* < 0.01).

Regardless of behavioral characteristics, comparatively lower rates of *T. vaginalis* infection occurred in women using condoms during sex (13.40%, *P* < 0.05) and in women who had no sex recently (3.85%, *P* < 0.05). There were significant findings linking *T. vaginalis* prevalence and the frequency of vulva cleaning (*P* < 0.05). Patients who bathed their vulva region more often than once a week were less likely to be *T. vaginalis* positive than those who did not (18.46% vs. 30.86%, *P* < 0.05). Similarly, the odds of *T. vaginalis* infection were lower in subjects with normal vaginal cleanliness grade I-II than in subjects with grade III-IV (1.01% vs. 27.36%, *P* < 0.05).

The individual symptoms, such as itching, abnormal odor, vaginal discharge, abdominal pain, and frequent urination, were associated with *T. vaginalis* infections. Only 7.11% of the asymptomatic women were positive for *T. vaginalis*, which was significantly lower than 33.97% in symptomatic group (*P* < 0.01). None of the women who currently have VVC was infected with *T. vaginalis*. Being diagnosed positive for VVC was strongly associated with *T. vaginalis* (*P* < 0.01), while BV was not (*P* > 0.05).

Based on the multivariate adjustment (Table 2), women who reported clinical symptoms were more than four times as likely to acquire *T. vaginalis* infections (OR = 4.58, 95% CI: 2.32–9.04, *P* < 0.01). III–IV vaginal cleanliness was also identified as an independent risk factor for *T. vaginalis* infections (OR = 29.71, 95% CI: 3.95–223.56, *P* < 0.01). Tertiary education (OR = 0.36, 95% CI: 0.16–0.81, *P* < 0.01) and yearly family income > 100,000 yuan (OR = 0.48, 95% CI: 0.26–0.91, *P* < 0.05) were protective factors against being *T. vaginalis* positive.

## Discussion

Although *T. vaginalis* has been confirmed to be linked to a range of adverse gynecological and obstetric outcomes, little emphasis has been placed on detecting its prevalence and risk factors in the recent past, such that there is no overall prevalence rate in China. This population-based study is the first to compare the prevalence rate of *T. vaginalis* in two hospitals located in different geographical regions (central vs. western China). A relatively high rate (20.94%) of *T. vaginalis*-infected females were identified in the Tibetan area compared with those (2.84%) obtained in Wuhan city. The prevalence rate of *T. vaginalis* in Wuhan city, a city with relatively richer medical resources in central China, is close to the reported rates from other regions (range from 0.4 to 7.4%) in China. This study focused on the possible risk factors in the female population in the Tibetan area. Among this population, risk factors related to *T. vaginalis* infection include lower educational status and family income, clinical symptoms, and a high level of vaginal cleanliness.

*T. vaginalis* was present in 20.94% of these Tibetan patients, which was obviously higher than that in the WHO regions (range from 1.60 to 11.70%) (Rowley et al. 2019) and in other reported provinces of China (Guangxi, 0.38%; Henan, 1.64%; Sichuan, 2.52%; Shandong, 2.80%; Fujian, 3.17%; Jilin, 7.44%) (Chen et al. 2006; Dai et al. 2010; Fang et al. 2007; Li et al. 2008; Lu et al. 2022; Zhang et al. 2018). It is a comparable rate of *T. vaginalis* to that previously reported among HIV-positive pregnant women (20.14%) in South Africa (Peters et al. 2021). However, in South Africa, a systematic review including 48 studies from 2015 to 2020 reported a pooled prevalence of 13.8% among pregnant women (Nyemba et al. 2021). This illustrates that the prevalence of *T. vaginalis* varies based on population characteristics. Although the disparity between the two regions presented above might be due to differences in the target population, this result indicated the widespread existence of *T. vaginalis* infection in the Tibetan area. Although wet-mount microscopy showed a poorer sensitivity (Ghallab et al. 2021) for detecting *T. vaginalis* in the Tibetan area than nucleic acid hybridization, which was used in Wuhan city, the result was unexpected. Given the low sensitivity of wet-mount microscopy, it is likely that the actual prevalence rate of *T. vaginalis* may be higher than that in the present study. Thus, identifying socioeconomic status and behavior and lifestyle factors rather than the population specificity of selected Tibetan women might be crucial to explain the *T. vaginalis* prevalence.

Furthermore, it is well established that *T. vaginalis* infection is a sexually transmitted infection (Workowski and Bolan 2015). Inadequate treatment and lack of concurrent treatment of sexual partners may be related to the continuing epidemics of *T. vaginalis* infection (Schumann and Plasner 2022). As our findings shows, the prevalence of *T. vaginalis* infection was highest in the Tibetan area, followed by BV and VVC. This result was inconsistent with some previous reports including another Tibetan area in China and a resource-limited region in India, both of which showed that *T. vaginalis* was less common than BV or VVC (Dai et al. 2010; Khan et al. 2019). Lack of formal treatment for *T. vaginalis* infection seems to be a reason for the disparity, leading to a high prevalence.

In this study, women with educational levels lower than college were more likely to have a *T. vaginalis* infection, which is consistent with early findings from the USA and Nigeria (Aboyeji and Nwabuisi 2003; Rosenbaum 2018; Tompkins et al. 2020). As a result of lower education, women may lack knowledge of *T. vaginalis* or other sexually transmitted diseases; thus, they may be unaware of the importance of correct, habitual, and consistent use of condoms. Among the highly educated women in the current study, nearly half (46.34%) reported condom use with regular partners; in contrast, a low rate of condom use (11.30%) was shown in the primary or below education subgroup. Another potential reason for this issue is that low-educated women do not realize the need to seek medical attention upon experiencing symptoms of *T. vaginalis* due to limited knowledge about the disease. Such conditions might increase the spread of *T. vaginalis*. Thus, further efforts to improve educational levels are needed to prevent *T. vaginalis* infection in the Tibetan area.

Family income was strongly associated with the likelihood of *T. vaginalis* infection in our study. Cases were more prevalent among females with family income below the average level, which is the same as prior reports in the USA and southern Brazil (Gatti et al. 2017; Patel et al. 2018). In accordance with the WHO, women in low-income countries undertake a greater burden of *T. vaginalis* infection (Organization. 22 August 2022). For one, lower income in many cases means lower educational attainment, the evidence of which was a smaller proportion (15.40%) of women with lower income received a college education compared to women with higher family income. Another reason for this is poor access to health care in low-income women, which leads to a higher risk of transmission of *T. vaginalis*. Thus, more interventions for improving living standards should be targeted to this population.

Using a condom for sex was a protective factor against *T. vaginalis* infection, as reported by Barbosa et al. (2020). In this study, women who did not use condoms for sex were twice as likely to be *T. vaginalis* positive than women who did. Speculatively, we would suggest that the marked disparity might be partly due to continued transmission from asymptomatic sex partners. Without awareness of infection, asymptomatic sex partners readily transmit *T. vaginalis* to women during penile-vaginal sexual encounters. Additionally, women who reported no sex in the past six months were less likely to be infected with *T. vaginalis*, which is closely linked to a reduction in the likelihood of transmission via sexual contact. These results highlight that consistent and correct condom use can prevent the transmission of *T. vaginalis* by providing an effective barrier against pathogens (Wan Muda et al. 2018).

The proportion of asymptomatic carriers of *T. vaginalis* should not be overlooked. Our study found that only 14 of the 85 (16.47%) *T. vaginalis*-positive women were asymptomatic, compared with nearly 80%, a universally recognized level (Workowski and Bolan 2015). A greater percentage of *T. vaginalis*-positive women came to this hospital because they had related uncomfortable symptoms. This is problematic since some women in this area are unaware of the importance of having regular gynecological examinations unless symptoms appear. Hence, general surveillance for sexually transmitted diseases, an effective way to prevent *T. vaginalis* infection, is needed.

The major strength of this study was the high rate of testing *T. vaginalis* infection and a real-life condition in the process of diagnosis in the hospital. Moreover, the characteristics of the population enrolled in this study have rarely been studied before. The limitations of the study were as follows. This study recruited only participants with associated symptoms or those who just came for medical attention in the hospital; thus, the limit affected the generalizability and led to selection bias. For measurement bias, our subsequent studies should introduce more sensitive tests and biochemical indicators based on direct microscopy to improve the sensitivity.

On the basis of this study, we conclude that the prevalence of *T. vaginalis* infection is estimated to be 20.94% among women treated in the hospital of Shannan city during this period. Associated risk factors included lower educational status and family income, higher sexual frequency, nonuse of condoms, clinical symptoms, and a high level of vaginal cleanliness. Our results have improved our understanding of the prevention of *T. vaginalis* in the Tibetan area. To effectively reduce the occurrence of *T. vaginalis* infection in this area, adopted measures depended on different customs and demographic characteristics, including raising the standard of living as well as women’s educational level and promoting reproductive hygiene habits.

## Supplementary Information

Below is the link to the electronic supplementary material.

Supplementary file 1 (XLSX 69 KB)

Supplementary file 2 (XLSX 30 KB)

## Data Availability

All relevant data are within the paper.
